# Contacting Layer Affects Properties of Piezoelectric Poly-L-Lactide Biomaterial

**DOI:** 10.3390/polym18020257

**Published:** 2026-01-17

**Authors:** Marija Vukomanovic, Martina Žabčić, Lea Gazvoda, Marija M. Babić Radić, Simonida Lj. Tomić

**Affiliations:** 1 Advanced Materials Department, Jozef Stefan Institute, Jamova 39, 1000 Ljubljana, Slovenia; martina.zabcic@ijs.si (M.Ž.); lea.gazvoda@ijs.si (L.G.); 2 Jozef Stefan International Postgraduate School, 1000 Ljubljana, Slovenia; 3 Innovation Center, Faculty of Technology and Metallurgy, University of Belgrade, Karnegijeva 4, 11000 Belgrade, Serbia; mbabic@tmf.bg.ac.rs; 4 Faculty of Technology and Metallurgy, University of Belgrade, Karnegijeva 4, 11000 Belgrade, Serbia; simonida@tmf.bg.ac.rs

**Keywords:** contacting layers, Teflon, Teflon S, polyimide, paper, organic piezoelectric, poly-l-lactide, piezostimulation, human keratinocytes

## Abstract

The main limitations of using a high-temperature drawing approach to tailor poly-l-lactide (PLLA) crystallization and molecular orientation for ultrasound-active piezoelectric structures stem from the intrinsic properties of the processed polymer, including low melting/softening elasticity and slow crystallization kinetics. Here, we found that applying different contacting layers, including polytetrafluoroethylene (PTFE) (as Teflon and Teflon S), cellulose (Paper) or polyimide (Kapton) deposited at the surface of PLLA, significantly affects the drawing process and tailors its oriented crystallization and molecular chain orientation. Consequently, the contacting layers contribute to the piezoelectric properties of PLLA (alone or with added morphologically anisotropic hydroxyapatite (HAp) filler), affecting its activation via ultrasound and generated electro-signal. Human keratinocytes (HaCaT cells) stimulated on these surfaces are shown to receive and respond to the transferred stimuli via the activation of the cytoskeleton and directional migration. The high-temperature (250 °C) drawing approach with contacting layers is a simple, solvent-free and economically viable way of broadening the applications of classical high-temperature drawing, opening new possibilities for further tailoring the piezoelectricity of organic piezoelectrics.

## 1. Introduction

Recently, poly-l-lactide (PLLA) organic piezoelectric has garnered increasing interest as a promising naturally sourced biomaterial for designing next-generation stimuli-active medical devices [1,2,3,4]. This increased interest partially stems from the biodegradable and biocompatible nature of PLLA, approved by the European Medicines Agency (EMA) and Federal US Food and Drugs Administration (FDA) for use in therapeutics and medical devices [5], which has been validated during extensive investigations over the past few decades and through clinical applications [6,7,8]. Indeed, biodegradable organic piezoelectric PLLA is potentially applicable for implantable bioelectronics, advanced biomedical sensing technologies, stimuli-responsive drug delivery and bioactive tissue regeneration approaches [2,4]. One particular advantage of using PLLA as an organic piezoelectric is its ability to be activated using ultrasound (US) [9]. This opens up the possibility of remotely activating the implanted biomaterial and triggering electrical stimuli on request, enabling minimally invasive, very precise and wireless electrical stimulation [2,10].

As an organic piezoelectric with a piezoelectric constant d_14_ = 9.82 [11], PLLA has a pivotal position among leading biodegradable piezoelectrics [1,12,13,14]. Nonetheless, its piezoelectric properties are low to enable effective applications and require further tailoring [4,13,14,15]. For that purpose, diverse strategies based on processing composites, applying fillers (including morphologically anisotropic hydroxyapatite (HAp) crystalline particles), and physical and chemical modifications were developed [1,4,14,15,16]. Due to the helix structure with polar C=O groups bonded to the asymmetric C-atom in PLLA chains, the orientation of dipoles is random, resulting in zero net polarization [14,15]. Shearing the PLLA helices slightly orients the C=O dipoles, leading to net polarization and inducing shear piezoelectricity [14,15]. This type of structure allows all processing approaches to focus on increasing the PLLA piezoelectricity: these approaches are either performed in solid, solution or melt form or target two critical parameters, namely crystallization (crystalline phase, degree of orientation and crystallite orientation) and molecular chain orientation [1,14].

Processing the polymer melt or softened solid enables controlled solidification of the polymer under unidirectional force, which affects its structural properties and enables the creation of oriented semi-crystalline structures [1]. The main advantages of processing PLLA from melt or softened solid are its simplicity and avoiding the use of toxic organic solvents. However, the main challenge with this type of processing remains keeping the molecular chain orientation stable as the polymer melts and the softened solid relaxes rapidly [14]. The main commonly controlled parameters are the annealing temperature, drawing rate and drawing ratio (DR) [4], which are found to be 80–90 °C, 40 mm/min and DR5 for PLLA [17]. The stretching, however, strongly depends on the molecular chain’s mobility in the melt and softened solid, the mechanical properties of the polymer and the effect of the force used for mechanical drawing. At a certain point, the drawing ratio reaches the maximum possible value that it can achieve without breaking the film, which also limits the maximal contribution of applied force.

In general, PLLA suffers from low melting and softening strength, poor thermal resistance, low crystallization kinetics and brittleness, which is particularly increased as the percentage of crystallinity increases [18]. Fortunately, there are processing approaches, including branching, adding fillers and blending with other polymers, that can increase its melting and softening strength and crystallization behavior. In particular, it has been observed that PLLA composites, containing low contents of polytetrafluoroethylene (PTFE) or cellulose blended with PLLA, can improve crystallization and mechanical properties, including improving melting elasticity [19,20]. Interestingly, tailoring the properties of PTFE tailors its influence to fit the crystallization and mechanical properties of another polymer, which then allows for applying different types of PTFE as another processing option [21].

Despite recent progress, optimizing the piezoelectricity of PLLA still remains one of the important challenges to overcome for achieving the full potential of biodegradable flexible bioelectronics. Starting from the above-described benefits and limitations of high-temperature drawing for optimizing the piezoelectricity of PLLA, this study explored the applicability of another polymer, deposited at the surface of PLLA as the contacting layer, and its potential contribution to the drawing process for achieving improved crystallization, molecular orientation and increased piezoelectricity. For the very first time we investigated the use of the contacting layer as a tool for tailoring piezoelectricity of the PLLA and hypothesized that piezo-PLLA films with different contacting layers would be differently activated via US, which would affect the piezostimulation of cells directly grown at their surface and their response to transferred electro-stimuli.

## 2. Materials and Methods

### 2.1. Materials

Poly-L-lactide resomer L 207 S (Evonik, Essen, Germany), chloroform (≥99.8%, Sigma-Aldrich, St. Louis, MO, USA), N,N-Dimethylformamide (DMF, anhydrous, 99.8%, Sigma Aldrich), Polytetrafluoroethylene (PTFE, Teflon, Dastaflon, Madvode, Slovenia), Teflon S (Coating Solutions, Hugo, MN, USA), Polyimide (PI, Kapton, Dasteflon), Baking Paper, dihydrorhodamine 123 (DHR, >95%, Sigma-Aldrich), Dulbecco’s Modified Eagle Medium (DMEM, Sigma-Aldrich), fetal bovine serum (FBS, Gibco, Waltham, MA, USA), penicillin–streptomycin (1:1, Gibco), Dulbecco’s Phosphate-Buffered Saline (DPBS, Sigma-Aldrich), TrypLE select (Gibco, Waltham, MA, USA), poly-L-lysine solution (mol wt. 70,000–150,000, 0.01%, sterile-filtered, Bio Reagent, Sigma, Hoddesdon, UK), the Presto Blue Cell Viability Reagent (Molecular Probes, Thermo-Fisher Scientific, Waltham, MA, USA), the bicinchoninic acid (BCA) protein detection assay kit (Sigma Aldrich), RIPA Lysis and Extraction Buffer (Thermo Scientific, Waltham, MA, USA), the HaltTM protease and phosphatase inhibitor single-use cocktail (100×) (Thermo Scientific), Triton X 100 (Sigma-Aldrich Life science, St. Louis, MO, USA), paraformaldehyde (Sigma-Aldrich, Hamburg, Germany), Rhodamine Phalloidin (RP, Invitrogen by Thermo Fisher Scientific, Waltham, MA, USA), and DAPI (diamidino-2-phenylindole, Biotium, Fremont, CA, USA) were used.

### 2.2. Processing and General Characterization

Processing PLLA films: After dissolving PLLA (2 g) in chloroform (20 mL), 5 mL of DMF was added and the mixture was homogenized via mixing with a magnetic stirrer for the next 2 h. If HAp filler was added, it was dispersed in DMF to form 1 wt.% PLLA HAp. The HAp filler was synthesized using a previously developed sonochemical approach [22,23]. It was dispersed in DMF, and the mixture was added to the polymer solution to homogenize. The mixture was solvent-casted in a Petri dish (φ = 15 cm) and dried polymer was further processed into films, as previously performed in [17]. The polymer was placed into a rectangle aluminum mold covered by a contacting layer (Teflon, Teflon S, PI or Paper), pressed via metallic plates and then heated to 250 °C for melting inside the mold. Afterwards, it was hot-pressed (40 kN, 2 min) and immediately quenched in water (10 °C) to form non-drawn films (PLLA DR1 or PLLA HAp DR1). The DR1 films were pre-heated at 80 °C and then uniaxially drawn (with 40 mm/min rate) to 5 times their initial length to form PLLA DR5 and PLLA HAp DR5 films. Depending on the type of contacting layer used, the films were labeled PLLA DR5 Teflon, PLLA DR5 Teflon S, PLLA DR5 Paper or PLLA DR5 PI, as well as PLLA HAp DR5 Teflon, PLLA HAp DR5 Teflon S, PLLA HAp DR5 Paper or PLLA HAp DR5 PI.

Structural characterization: The crystalline phases were identified via X-ray diffraction analysis using EMPYREAN (2θ range 10–60°, 0.026° step size with a 500 s time of capture). Small- and wide-angle X-ray scattering (SAXS and WAXS) analyses were performed via a SAXS Point 2.0 system (Anton Paar, Institute of Biotechnology of the Czech Academy of Sciences, BIOCEV Centre, Vestec, Czech Republic) equipped with a Gallium source (λ = 1.34 Ä), a synchrotron, and an Eiger R 1 M detector at room temperature in transmission mode. Intensities were taken for a 180 ms frame rate and a 15 s acquisition time. Differential scanning calorimetry (DSC) (NETZSCH STA 449), performed in an Ar/O atmosphere (40/10), was used to determine the total crystallinity of films. The samples were measured in a range from 40 °C to 200 °C at a 20 °C/min heating rate, and the percentage crystallinity (Xc) was calculated using Xc (%) = ((∆H(m) − ∆Hcc))/(∆H100), with ΔH100% corresponding to theoretical 100% crystalline PLLA films with α crystalline form (93.6 J/g). Polarized Raman spectrometry (NTEGRA Spectra NT-MDT) was used to determine the molecular chain orientation of the polymer. The spectra were recorded using a 488 nm laser in the range 200–3200 cm^−1^. Normalization was carried out to the CH_3_ bend (1454 cm^−1^), and the order ratio (R) was calculated as the ratio of the C–COO stretching bend (875 cm^−1^) intensities for the parallel and normal orientation of the polarizer relative to the direction of film drawing.

Surface properties: X-ray photoelectron spectroscopy (XPS, K-alpha, Thermo VG Scientific, East Grinstead, UK) was performed to determine the surface chemistry. Measurements were performed using a monochromated Al source operated at 1486.6 eV, 12 kV, and 3 mA, and the 6.8 × 10^−7^ Pa working pressure and charge shifting were corrected based on the C1s (285.26 eV) and O1s (532.29 eV). Data were analyzed using Spectral Data Processor software (ver. 4.3). Wettability was investigated using the Theta Lite contact angle meter system (Biolin Scientific, Västra Frölunda, Sweden). The water contact angle (WCA) was measured using the sessile drop method, through which a drop of milli-Q water (5 μL) was placed onto the film surface (10 × 10 mm) and recorded to determine the contacting angle. The WCA results were the mean values of at least three measurements. The surface morphology of the films was investigated using scanning electron microscopy (JSM-7600 F, Jeol Ltd., Tokyo, Japan).

Piezoelectric properties: The piezoelectric response was measured as the voltage output generated during mechanical deformation using US. Deformation was generated via a 1 MHz US transducer (Mfd. Sun Medisys, Delhi, India, Deltasound 1 MHz) using a US-conductive hydrogel (Ultragel Hungary 2000 Kft., Budapest, Hungary) or via an 80 kHz US ultrasonic bath (80 kHz, Elma, Elmasonic P, Singen, Germany) filled with water. Measurements were performed for copper electrodes at two positions: on the top and bottom of the film and on the side edges of the film; these positions corresponded to their drawing directions and generated voltages, as detected using a Kaysight (Rosebery, Australia) MSOX3034T oscilloscope. The measurements were performed for at least three different films.

Reactive oxygen species (ROS): A dihydrorhodamine 123 (DHR) assay [24] was applied for detecting ROS generation by films with different contacting layers before and after activation via US. The films (5 × 5 mm size) were placed into 96-well plates containing physiological solution (0.9% NaCl). Testing included four 96-well plates with the same set of samples. Two plates were stimulated with 1 MHz US, while the remaining two were not stimulated. One each of the stimulated and non-stimulated plates received vitamin C as an ROS scavenger (40 µL/well, 1 mg/mL). In addition, the negative control was NaCl medium, while the positive control was H_2_O_2_ (5 wt%) pre-irradiated with UV light. After adding the DHR agent (10 µL per well, 0.1 mM) following US stimulation, the samples were incubated for 1 h at 37 °C and then measured to determine the fluorescence at 490/530 nm Ex/Em. All samples were tested in triplicate.

### 2.3. Interactions with Human Cells

Human cell piezostimulation: Low-passage HaCaT human keratinocytes (ATCC PCS-200-011) were grown in 6-well plates in full DMEM (with 10% FBS and 1% penicillin–streptomycin). PLLA films with different contacting layers (1 × 1 cm size) were immersed in poly-L-lysine solution (300 µL), washed with full DMEM and then seeded with HaCaT cells inside 24-well plates (20.000 cells/well). Two 24-well plates with the same set of samples were further incubated at 37 °C and 5% CO_2_ (MCO-19AIC(UV)-PE, Panasonic). After incubation, the films with adhered cells were replaced in new 24-well plates, and cell attachment was measured using the Presto Blue Cell Viability Reagent (following the provided protocol). The reagent was washed with DPBS, and the plates were filled with fresh DMEM. Stimulation was conducted according to a previously optimized protocol [16]. One of the plates was stimulated via 1 MHz US, while the second plate was not stimulated. The stimulated plate was immersed in the Petri dish (20 cm diameter) filled with 100 mL of distilled water, which was connected to the 1 MHz US transducer via a thin layer of US transducing gel. The stimulation was carried out using 1:10 on–off intervals and 1.8 W/cm^2^ of power. Both plates were then transferred to a CO_2_ incubator, and incubation was performed for the next 24 h. The stimulation and Presto Blue growth detection steps were repeated daily for the next 72 h. Analysis was performed in at least two independent experiments, repeated each time in three parallel experiments.

Total protein detection: The bicinchoninic acid (BCA) assay was applied for quantifying the total protein levels following the protocol for the 96-well plates. The protein levels were detected in cells adhered to the surface of films, as well as in cells that proliferated on films for 72 h with or without piezostimulation. The total cell lysates were prepared by incubating films with cells in RIPA lysis buffer supplemented with enzyme inhibitors, scratching film surfaces to detach cells, separating the suspended cells/cell debris and incubating them at low temperature, and performing sonication and centrifugation. All samples were tested in triplicate.

Actin filament detection: Actin expression was detected in cells grown on the surfaces of films with different contacting layers with or without stimulation. Detection was performed in the final step after 72 h of stimulation and growth. The cells were fixed with paraformaldehyde (3.7% solution in DPBS), permeabilized with Triton X 100 (0.5% solution in DPBS), washed with DPBS, and finally stained with RP (1 µL/mL) for actin and DAPI (5 µL/mL in Hank’s balanced salt solution) for nuclei detection. The stained cells on top of the films were observed under a fluorescence-inverted microscope (Eclipse Ti-U Nikon). Analysis was performed for three parallel samples and in at least two individual experiments.

Cell morphology detection: The morphological properties of HaCaT cells adhered and grown on the surfaces of films with different contacting layers with or without US stimulation were observed using SEM microscopy. Cells were fixed in glutaraldehyde (2.5 w% in DPBS) for 2 h at room temperature. After fixation, they were washed with DPBS and then gradually dehydrated using a series of ethanol dilutions (30%, 50%, 70%, 80%, 100%). In each of the ethanol solution films, cells were incubated for 10 min, and in the final 100% ethanol solution, they were left for 30 min. The last step was chemical drying, which was performed by incubating samples in 100% HMDS for 15 min, before replacing the HDMS with fresh drying agent, which was then left to dry in air. Samples were sputtered with gold before examination via SEM (JSM-7600 F, Jeol Ltd., Akishima, Japan).

Statistical analysis: The results are presented as the mean values of at least three measurements (*n* = 3) and are indicated via standard deviation (SD). Differences between groups were assessed via one-way ANOVA (GraphPad 9.0 Software) with a confidence level of 95% and *p* < 0.05.

## 3. Results

**Figure 1:**
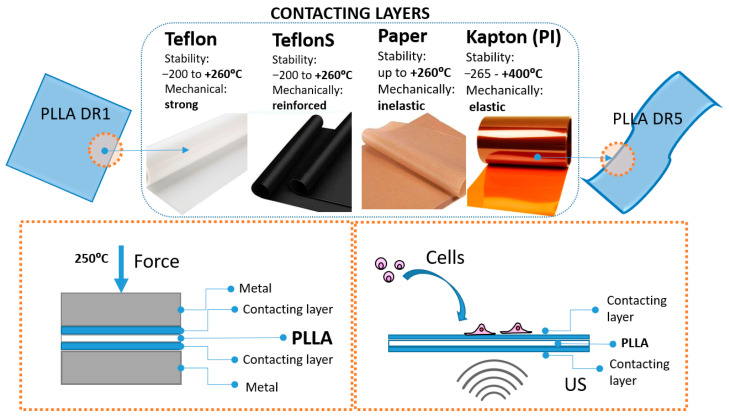
The use of different contacting layers for processing uniaxially drawn PLLA films to design their piezoelectricity and tailor the ultrasound-activated piezostimulation of human cells.

Processing PLLA into uniaxially drawn films, characterized by piezoelectric properties, always starts by hot pressing the polymer into non-drawn films (DR1). Hot pressing is performed by placing the polymer powder inside an aluminum mold between two metallic surfaces. To more easily detach the hot-pressed PLLA DR1 film from hot metallic surfaces, another inert protecting layer is commonly applied. This protecting layer, labeled here as the contacting layer, should be thermally resistant: it is quenched in cold water along with the PLLA DR1 films and then physically separated from its surface. Here, we investigated the influence of different contacting layers, including Teflon, Teflon S, Kapton (PI) and Paper, on the surface chemistry of the final processed PLLA DR5 films, their potential role in crystallization and structural properties of piezo-PLLA and their influence on the cells stimulated directly at the surface of these films, as illustrated in Figure 1.

### 3.1. Surface-Induced Crystallization of PLLA

**Figure 2:**
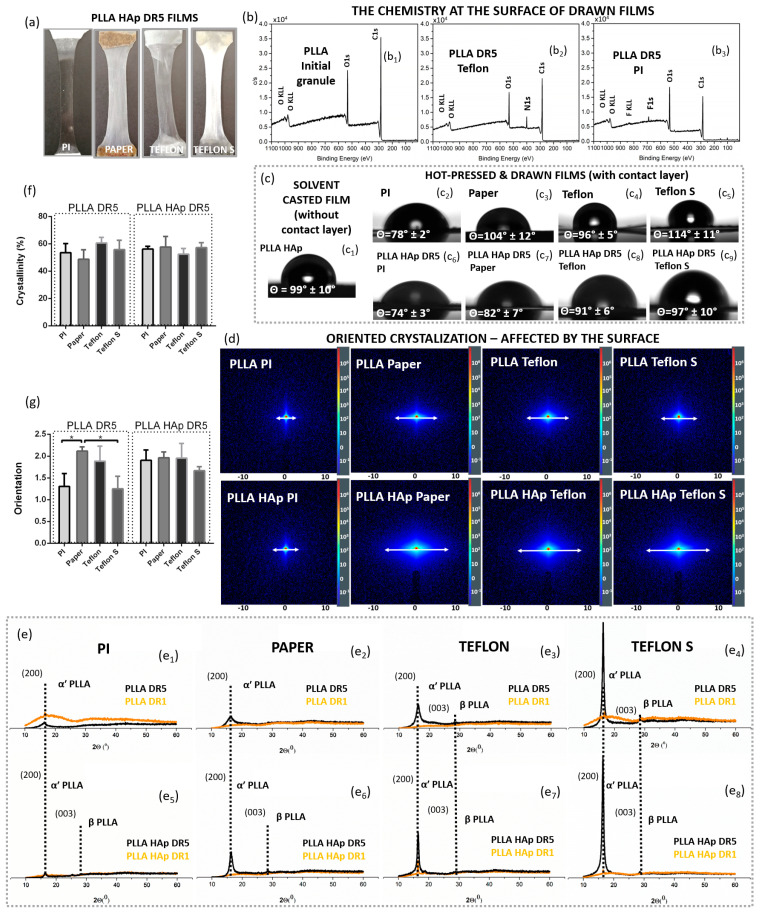
The influence of surface chemistry on PLLA crystallization. The physical appearance of the PLLA HAp DR5 films pressed using different contact surfaces (Paper, Teflon, Teflon S and PI) (**a**), the XPS spectra of non-processed PLLA (**b_1_**) and PLLA processed with Teflon (**b_2_**) and PI (**b_3_**), confirming their presence at the surface; the water contact angles of PLLA without a surface layer (**c_1_**) and pairs of materials used as contacting layers (Paper, Teflon, Teflon S and PI (**c_2_**–**c_5_**)) and relative PLLA HAp DR5 films (**c_6_**–**c_9_**); SAXS patterns showing differences in the crystallization of PLLA and PLLA HAp films with different contacting layers (**d**); the XRD patterns of hot-pressed (DR1) and drawn (DR5) PLLA (**e_1_**–**e_4_**) and PLLA HAp (**e_5_**–**e_8_**) films with different contact layers at the surface; and DSC determined the crystallinity (**f**) and polarized Raman spectroscopy determined the orientation, * refer to *p* < 0.05 (**g**) of the PLLA and PLLA HAp drawn films with different contacting layers at the surface.

After applying different contacting layers (PI, Paper, Teflon or Teflon S) when melt pressing PLLA and PLLA HAp into DR1 films, the resulting films had macroscopically different appearances (Figure 2a). The XPS spectra confirmed the F1s peak at the surface of PLLA processed with Teflon and Teflon S (corresponding to F in C-F bonds at 689 eV) [25] and the N1s peak (corresponding to C-N at 400.1 eV) [26] at the surface of PLLA processed with PI, as well as their absence in the XPS spectrum of non-processed PLLA granules. Based on these differences in surface chemistry, we suspected that thin depositions of contacting layers remained on top of the processed films (Figure 2b).

To further evaluate the surface, we investigated the wettability of films and confirmed that, in addition to the surface chemistry, different contacting layers affected the WCAs of the processed films. The initially hydrophobic surface of the PLLA film was changed after applying the contacting layers, which confirmed their presence on the film surfaces (Figure 2c). In particular, PLLA HAp films processed with the PI contacting layer had practically the same WCA as PI itself. The low standard deviation (SD) identified for consecutive measurements along the film indicates its uniform coverage by PI. On the other hand, Teflon, Teflon S and Paper contacting layers did not change the initial WCA of PLLA so intensively, and high SDs for different measurements pointed to variations in wettability due to the partial coverage of certain areas.

Next, we investigated the potential contributions of contacting layers to the structural properties of PLLA films. According to the 2D SAXS data, the presence of the top layer significantly affected the structure of the polymer in the PLLA and PLLA HAp films (Figure 2e). The lower scattering around the central beam obtained for films with PI at the top indicated low long-range ordering, which was not notably increased in the presence of the HAp filler. If other contacting layers were present on top of the PLLA films, scattering was significantly intensified, and it was further enhanced if an HAp filler was present. While the intensity of scattering indicated long-range ordering, the shape of the diffuse rings in 2D SAXS revealed that the PLLA had a crystallite orientation. The elongated diffuse rings obtained for films drawn with Paper, Teflon or Teflon S on top (Figure 2d) indicated preferentially oriented crystallization. In addition to enhancing long-range ordering, the presence of the HAp filler further enhanced preferential crystallization. Increased long-range ordering in the PLLA and PLLA HAp films with different contacting layers was detected for 1D SAXS (Figure S1).

The contribution of contacting layers on top of PLLA to crystallization has already been identified in initially formed DR1 films. In contrast to PLLA DR1 films with PI on top that were amorphous (with or without HAp filler), the XRD patterns of other films confirmed the initial presence of the α′ PLLA phase and its further crystallization during unidirectional drawing in PLLA DR5 films. For all crystallized films, (110)/(200) preferentially oriented crystallization was observed. Crystallization was enhanced from PLLA films with PI toward Paper to Teflon and Teflon S surface layers (Figure 2e). Additionally, the presence of a HAp filler encouraged the formation of the β PLLA phase. Interestingly, for the Teflon and Teflon S contacting layers, the β PLLA phase was detected in both the PLLA and PLLA HAp films (Figure 2e_3_,e_4_,e_6_,e_7_).

In contrast to the increased preferential orientation in the (200) direction, the total crystallinity based on DSC measurements did not show statistically relevant differences. Similarly, the total molecular chain orientation was statistically increased for PLLA DR5 films with Paper on top; however, these differences were lost in cases where the HAp filler was present.

**Figure 3:**
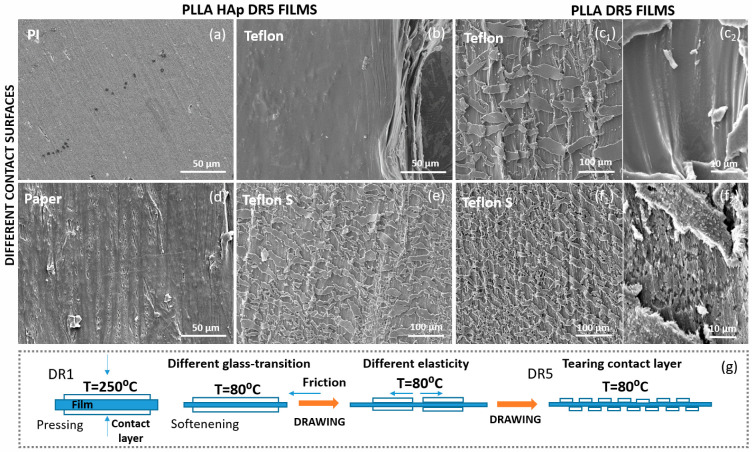
The influence of the contact layer on the surface morphology of PLLA. Changing the roughness at the surface of the PLLA HAp DR5 films with PI (**a**), Teflon (**b**), Paper (**d**) and Teflon S (**e**) at the top; the intensive roughness on the surface of the PLLA DR5 films with Teflon (**c_1_**,**c_2_**) and Teflon S (**f_1_**,**f_2_**); the adhesion of the contact layer to the surface of the DR1 film during processing and its tearing during film drawing to DR5 (**g**).

SEM investigations clearly confirmed the presence and morphological distribution of the contacting layer at the surface of the PLLA films (Figure 3). While the PLLA HAp DR5 films with PI at the top had very smooth surfaces, the roughness of the films with Paper and Teflon S increased. The films with Teflon on top varied depending on the presence or absence of HAp. The morphology explained the variations in the WCA along the films and confirmed the assumption about the smooth deposition of PI and the partial presence of other contacting elements on the PLLA films’ surfaces. The observed morphologies suggest that the contacting layers were not initially randomly deposited on some parts of the PLLA when processing the DR1 films. They were probably torn off during uniaxial drawing, as illustrated in Figure 3g.

### 3.2. Ultrasound Activation of PLLA Films with Different Contacting Layers

**Figure 4:**
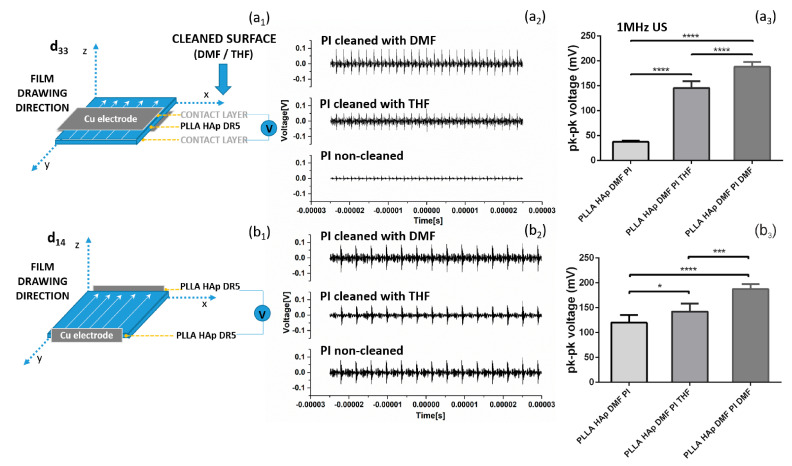
The piezoelectric properties of PLLA films with different surface contact layers. The voltage signal in the d_33_ direction (electrodes placed in direct contact with the contact layer) (**a_1_**), the piezoelectric properties of films during mechanical deformation via 1 MHz ultrasound (US) (**a_2_**) and the resultant voltage signals that depend on the type of contact layer (**a_3_**) and voltage signal in the d_14_ direction (electrodes placed on sides of the PLLA HAp films in direct contact with the PLLA HAp) (**b_1_**), and the piezoelectricity of the same films after mechanical deformation via 1 MHz US (**b_2_**) and uniform voltage signal (**b_3_**). Statistical analysis was performed for multi-comparisons, *n* = 4–5. *, *** and **** refer to *p* < 0.05, *p* < 0.001 and *p* < 0.0001, respectively.

The differences in surface layers were directly related to the ability of films to be activated via US. Depositing the electrodes at the top and the bottom of the films (as illustrated in Figure 4a_1_) provided significantly relevant variations in the detected output voltages, which depended on the type of contacting layer remaining at their surfaces (Figure 4a_3_). Despite these differences in intensity, all films were responsive to US activation (Figure 4a_2_). Obviously, there was a contacting layer in between the PLLA and the electrodes, which covered either the whole surface or just parts of it (as detected in the SEM images (Figure 3), therefore interfering with the detection of surface polarization and voltage output generation. In cases where the electrodes were deposited on edges of the films, which correspond to the film drawing directions (as illustrated in Figure 4b_1_), activation with US did not show very large variations between samples (Figure 4b_2_). There was a trend of increasing voltage output in the order PI < Paper < Teflon < Teflon S; these materials were deposited on the surfaces of films, which could be attributed to the contribution of these contacting layers to the oriented crystallization of the films, as seen in Figure 2.

### 3.3. Reactive Oxygen Species (ROS) Formation During Biomaterials Ultrasound Activation

**Figure 5:**
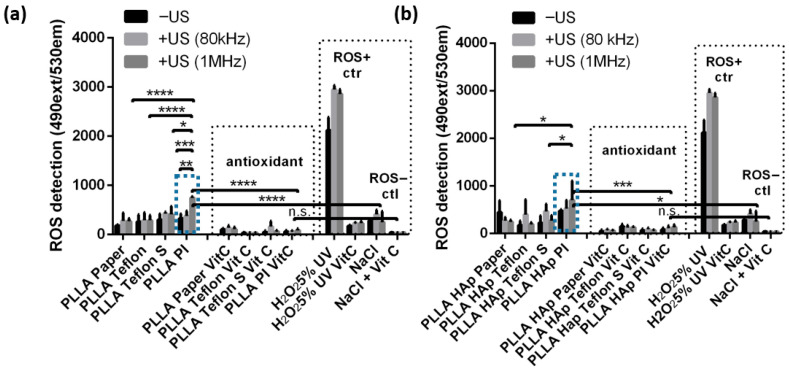
The influence of the contact layer on ROS formation during ultrasound activation. ROS formation detected for the PLLA (**a**) and PLLA HAp (**b**) films with Paper, Teflon, Teflon S and PI (marked with blue doted rectangle as only ROS active) contacting layers that were activated or not activated via US (80 kHz or 1 MHz). Vitamin C (Vit C)) was used as the ROS scavenger, while H_2_O_2_ (5%) UV and non-treated cells were used as positive and negative controls; statistical analysis was performed for multi-comparisons, *n* = 3. *, **, *** and **** refer to *p* < 0.05, *p* < 0.005, *p* < 0.001 and *p* < 0.0001, respectively.

The presence of contacting layers on the surface of PLLA films affected the ability of the films to produce ROS, particularly during mechanical activation via US (Figure 5). The production of ROS was measured using a DHR assay, and testing was performed for the same PLLA and PLLA HAp films, which differ only in the type of contacting layer, namely Paper, Teflon, Teflon S or PI. Testing was performed before and after activation via 80 kHz or 1 MHz US, and films and all controls were either activated via US or left without US treatment. As a detection control, the same set of films were tested with the addition of vitamin C (Vit C) as an antioxidant and ROS scavenger. In addition, the ROS positive control was H_2_O_2_ (5 wt%) pre-irradiated with a UV lamp (with or without Vit C), while the negative control was NaCl (with or without Vit C).

Looking at the controls (Figure 5a,b) revealed that DHR detected high levels of ROS in H_2_O_2_, and this level increased when treated with 80 kHz or 1 MHz US. A significantly lower level of ROS was detected in NaCl, and this level also slightly increased after activation via US. The high and low ROS levels detected in the positive and negative controls significantly declined in the presence of Vit C, confirming the validity of this method and quantitatively correlating the detected signal directly with ROS production.

The PLLA and PLLA HAp films with Paper, Teflon or Teflon S on the surface produced low levels of ROS, which were comparable to the levels of ROS produced by the NaCl solution, with slight variations depending on whether activation via US occurred. The exceptions were the PLLA and PLLA HAp films with PI on the surface. Initially, the ROS production of these films was low; however, after activation via US, it significantly increased (relative to films with other types of contacting layers (*p* < 0.05-0.0001) and relative to the NaCl-negative control (*p* < 0.0001)). A particular increase in ROS production for the PLLA PI and PLLA HAp PI films was achieved after activation via 1 MHz US. When Vit C was present, the ROS levels detected for all tested films decreased to a very low level that was statistically similar to the level of ROS produced by NaCl with Vit C.

### 3.4. The Interactions of Human Keratinocyte (HaCaT) Cells with Ultrasound Activated Biomaterials

**Figure 6:**
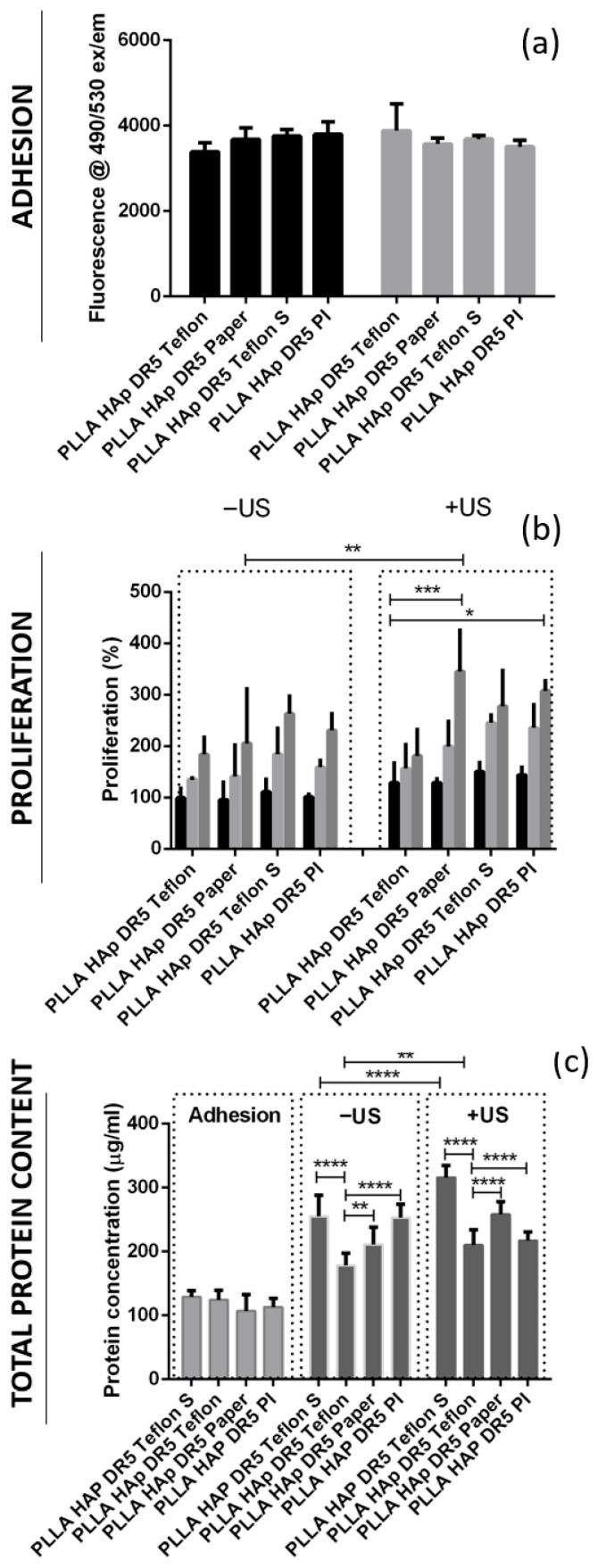
The stimulated growth of HaCaT cells on the surfaces of ultrasound-activated biomaterials. Cell adhesion (**a**), proliferation (24-, 36- and 48-h growth labelled in black, light gray and dark gray, respectively) (**b**) and total protein content (**c**) on the surfaces of the PLLA HAp DR5 films with different contact layers for treatment with or without US; statistical analysis was performed for multi-comparisons, *n* = 3. *, **, *** and **** refer to *p* < 0.05, *p* < 0.005, *p* < 0.001 and *p* < 0.0001, respectively.

Initially, the cells adhered on the films with different contacting layers (PI, Teflon, Teflon S and Paper) in a very similar manner, as observed based on the very similar levels of their metabolic activity on these surface (Figure 6a), as well as their very similar total protein contents measured from the lysates of cells extracted after adhesion on these surfaces (Figure 6c). The metabolic activity of cells after adhesion was measured for two sets of the same samples, as one of them was further activated via US while the second remained without US activation. Regardless of the type of contacting layer used in both sets, the differences in the cells’ adherence to the surface were statistically irrelevant. In this phase, the metabolic activity and total protein expression correlated with the number of adhered cells.

During the proliferation phase, cells adhering to films with different contacting layers (PI, Teflon, Teflon S and Paper) were analyzed for two cases: with and without activation via US. Without US activation, both metabolic activity and total protein content followed the same order (Teflon < Paper < PI < Teflon S), showing an increasing number of cells during proliferation on these surfaces (Figure 6b,c). HaCaT cells grew on the surface of PLLA HAp films with different contacting layers in the same fashion and without significant differences. However, when grown on top of the films activated with 1 MHz US, the differences started to appear (*p* < 0.005). Differences in metabolic activity (Teflon < Teflon S < PI < Paper) and total protein content (Teflon < PI < Paper < Teflon S) occurred, confirming the higher protein expression of cells grown on the surface of Teflon S, which was not only related to the increased number of cells grown on this surface but also encouraged another biochemical process potentially initiated via stimuli transfer from this surface during piezostimulation.

Direct comparison of the proliferation of cells on films with different contacting layers with and without activation via US showed interesting correlations. The metabolic activity of cells grown on surfaces with different contacting layers activated via US was always higher than that for those grown without US activation, and the same trend was identified for the total protein content, which was also statistically relevant (Figure 6b,c). The exception was films with PI on the surface, where protein content decreased.

**Figure 7:**
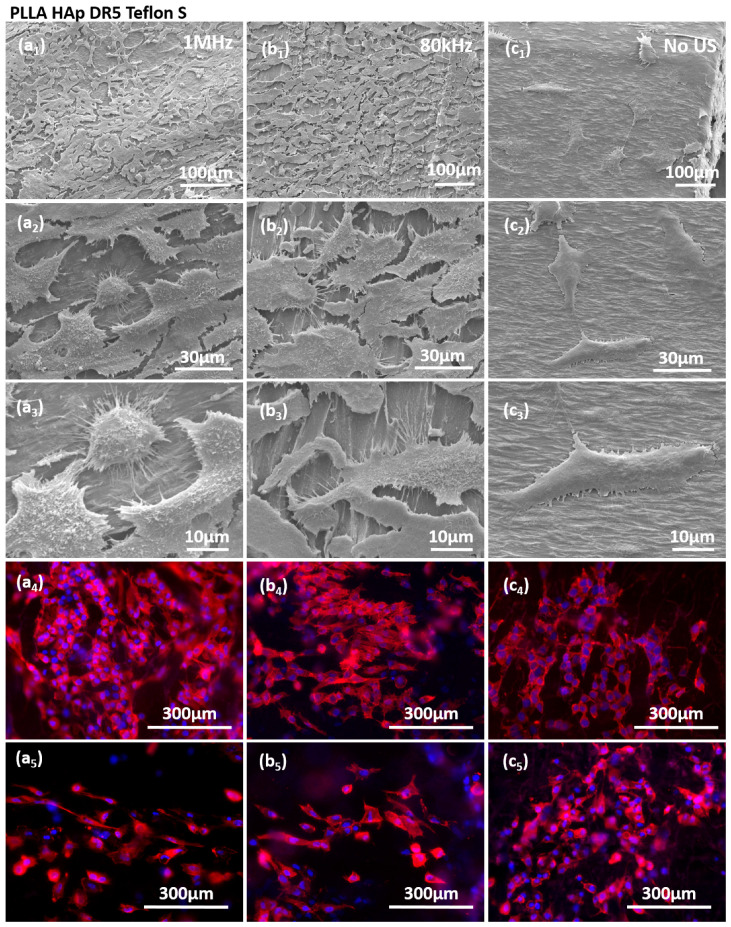
Morphology and actin filaments of HaCaT cells at the surface of PLLA HAp DR5 with Teflon S contacting layer stimulated with 1MHz US (**a_1_**–**a_5_**), 80 kHz US (**b_1_**–**b_5_**) and without US stimulation (**c_1_**–**c_5_**); red color refers to RP—stained actin and blue to DAPI- stained nuclei.

The stimulations of HaCaT cells on the surfaces of films with different contacting layers were further evaluated with regard to cell morphology and the expression of actin filaments (Figure 7 and Figures S2–S4). Cells grown on PLLA HAp films covered with different contacting layers, including Teflon S (Figure 7), Paper (Figure S2), Teflon (Figure S3) and PI (Figure S4), responded to US stimulation (either 1 MHz or 80 kHz) by increasing the cell density. Stimulated cells were morphologically elongated and oriented with increased expression of actin filaments, which indicated the activation of the cytoskeleton as a response to the stimulation. We also detected higher production of cellular protrusions, which cells used to connect to the substrate and to other cells. Interestingly, the cells favored the regions without the contacting layer, as clearly seen for PLLA HAp Teflon S (Figure 7b_3_), as the contacting layer could be morphologically detected as “islands” at the surface of PLLA and cells mainly occupied the area among them, which confirmed their preference for the stimulating structure and the role of direct contact between cells and the piezo-structure as an essential condition for transferring the signal during piezostimulation.

## 4. Discussion

A unidirectional mechanical force is frequently applied to the softened solid, melt or solution of PLLA to ensure oriented crystallization and enhanced molecular chain orientation—two essential parameters for obtaining a piezoelectric structure in the polymer [14,15]. For example, when adding a low content (1 wt%) of morphologically anisotropic fillers to the polymer bulk [16], the additives affect heterogeneous crystallization and act as nucleating and direction agents, which promote crystallization and direct the structural orientation.

A similar trend is observed for polymer blends, where adding a small amount of another polymer significantly affects the crystallization and mechanical characteristics of PLLA. As already proven, blending very low contents (below 10 wt%) of PTFE (Teflon) with PLLA provided significant mechanical reinforcement and toughening and increased elongation-to-break by 72%. PTFE also acted as a homogeneous nucleating agent, which promoted PLLA crystallization [19]. Similarly, blends of PLLA with low contents of cellulose (2 wt%) increased melting elasticity and promoted PLLA crystallization [20].

In contrast to alterations in the bulk, modifying the polymer surface and its potential effects on crystallization and structure orientation have been significantly less explored. Here, we observed that depositing a thin contacting layer on the surface of non-drawn PLLA films during hot pressing efficiently assists in enhancing the drawing force and contributes to oriented crystallization. Specifically, the contributions of the contacting layers come from their thermal and mechanical properties. As illustrated in Figure 1, the contacting layers used in this study all increased thermal stability: Paper, Teflon and Teflon S were stable up to 260 °C, and PI was stable up to 400 °C. The mechanical properties of the different contacting layers were characterized as follows: Paper was inelastic, Teflon and Teflon S were strong and reinforced, and PI was elastic. During the hot-pressing phase, Paper, Teflon and Teflon S were close to their thermal stability limits, and they were thermally deposited onto the PLLA surface. On the other hand, the more thermally stable PI was deposited mechanically due to its elasticity. Later, during the drawing phase, deposited layers were stretched along with PLLA films, which is when their elasticity had the highest contribution.

The lowest contributions to the structural properties of PLLA came from Paper and PI applied as contacting layers. Although they both effectively adhered to the PLLA surface, inelastic Paper was easily torn off in the very early phase of drawing, while highly elastic PI (higher than or equal to that of PLLA) was easily drawn together with PLLA. As these two contacting layers did not produce any additional force during unidirectional drawing, their contributions to the structural properties of PLLA were minimal.

Compared to PI and Paper, the contributions of Teflon and Teflon S were much greater. Previously, it was noticed that tailoring the properties of PTFE, by applying different PTFE types, replicates the influence that it has on other polymers within the blend (such as promoted mechanical strength, enhanced Young’s modulus, crystallization, etc.), as confirmed for the poly(butylene succinate)/PTFE blend [21]. Similarly, we applied two types of PTFE, Teflon and Teflon S, with two different mechanical strengths. As for PI and Paper, the adhesion of Teflon and Teflon S on top of PLLA was strong enough to remain stable on their surface during the drawing process. However, though their elasticity values were better than that of Paper, they were still much lower than those of PI and PLLA. Consequently, Teflon and Teflon S produced friction at the interface with PLLA, in the opposite direction to the drawing force. As Teflon S has been mechanically reinforced, the friction force produced with this layer was higher than that for the Teflon. At some point, the Teflon and Teflon S elasticity limit was reached, and they were torn off at the surface of the drawn PLLA. Morphologically, this produced repeating “islands” of the contacting layer that remained adhered in the drawing direction to the surface of the PLLA. The two opposite-side forces at the surface of the PLLA affected the structural properties of the drawn-induced crystallization of PLLA, including oriented crystallization and molecular chain orientation.

Previously, we observed that the presence of morphologically anisotropic fillers during the drawing process affects the phase composition of the crystallizing polymer and contributes to the low content of the β PLLA phase [16]. Interestingly, the same contribution has been detected for Teflon and Teflon S, for which oppositely directed drawing and friction forces at the interface between PLLA and the contacting layers also induced the partial crystallization of PLLA into the β PLLA phase. The contribution of HAp as morphologically anisotropic filler which improves oriented crystallization of PLLA is further affected by the type of contacting layer. Combined together anisotropic filler and contacting layer further tailor structural properties of PLLA and increase its piezoelectric properties.

As the contacting layer contributed to crystallization and orientation, consequently, these processes contributed to the piezoelectricity of PLLA. However, the presence of contacting layers at the surface was a barrier to the effective transfer of the signal during activation via US, as was clearly detectable when measuring the voltage outputs in two different setups, with and without the partial presence of contacting layers between the activated PLLA and measuring electrodes. A similar barrier existed during the piezostimulation of cells adhered to the top of the films with PLLA. It was very interesting to see cells migrating among the “islands” of contacting layers and reaching the area where they contacted the activated PLLA films. Here, they received the US-activated electro-stimuli, which increased the total protein content in cells and activated the cytoskeleton. This approach could be a very interesting strategy for directing the phases of cellular life and for applications in advanced regeneration and tissue engineering approaches.

## 5. Conclusions

Adding a contacting layer with optimal thermal and mechanical properties and the ability to strongly adhere to the polymer surface is a very effective new tool for tailoring the structural properties of uniaxially drawn films, optimizing their piezoelectric properties and, consequently, affecting their activation via US and interactions with human cells directly adhered to their surfaces. The approach could be tailored to optimizing and tailoring piezoelectric properties of other organic piezoelectrics, characterized by shear piezoelectricity.

## Data Availability

Data are available in repository Zenodo https://doi.org/10.5281/zenodo.17725263; https://doi.org/10.5281/zenodo.17725419; https://doi.org/10.5281/zenodo.17725392 (accessed on 26 November 2025).

## Supplementary Materials

The following supporting information can be downloaded at https://www.mdpi.com/article/10.3390/polym18020257/s1, Figure S1: 1D graph extracted from 2D SAXS images for PLLA and PLLA HAp films with different contacting layers; Figure S2. SEM morphology of HaCaT cells on the surface of PLLA HAp DR5 films with Paper as contacting layer (distribution of cells, connections among cells and cell attachments to the surface) obtained without ultrasound (US) stimulation (a1–3) as well as after stimulation using 80 kHz (b1–3) or 1 MHz (c1–3) US; cytoskeleton of cells observed without (a4,5) and with US (80 kHz (b4,5) or 1 MHz (c4,5)) US stimulation; Figure S3. SEM morphology of HaCaT cells on the surface of PLLA HAp DR5 films with Teflon as contacting layer (distribution of cells, connections among cells and cell attachments to the surface) obtained without ultrasound (US) stimulation (a1–3) as well as after stimulation using 80 kHz (b1–3) or 1 MHz (c1–3) US; cytoskeleton of cells observed without (a4,5) and with US (80 kHz (b4,5) or 1 MHz (c4,5)) US stimulation; Figure S4. SEM morphology of HaCaT cells on the surface of PLLA HAp DR5 films with PI as contacting layer (distribution of cells, connections among cells and cell attachments to the surface) obtained without ultrasound (US) stimulation (a1–3) as well as after stimulation using 80 kHz (b1–3) or 1 MHz (c1–3) US; cytoskeleton of cells observed without (a4,5) and with US (80 kHz (b4,5) or 1 MHz (c4,5)) US stimulation.

## Abbreviations

The following abbreviations are used in this manuscript:

PLLA	Poly-l-lactide
PI	Polyimide
PTFE	Polytetrafluoroethylene
ROS	Reactive oxygen species
HAp	Hydroxyapatite
DHR	Dihydrorhodamine 123
DMF	N,N-Dimethylformamide
DR	Drawing ratio
DMEM	Dulbecco’s Modified Eagle Medium
BCA	Bicinchoninic acid assay
