# Supplementary figures and images for: Contacting Layer Affects Properties of Piezoelectric Poly-L-Lactide Biomaterial
Source: Polymers (Basel). 2026 Jan 17;18(2):257. doi: 10.3390/polym18020257 (PMC12845684; doi:10.3390/polym18020257)

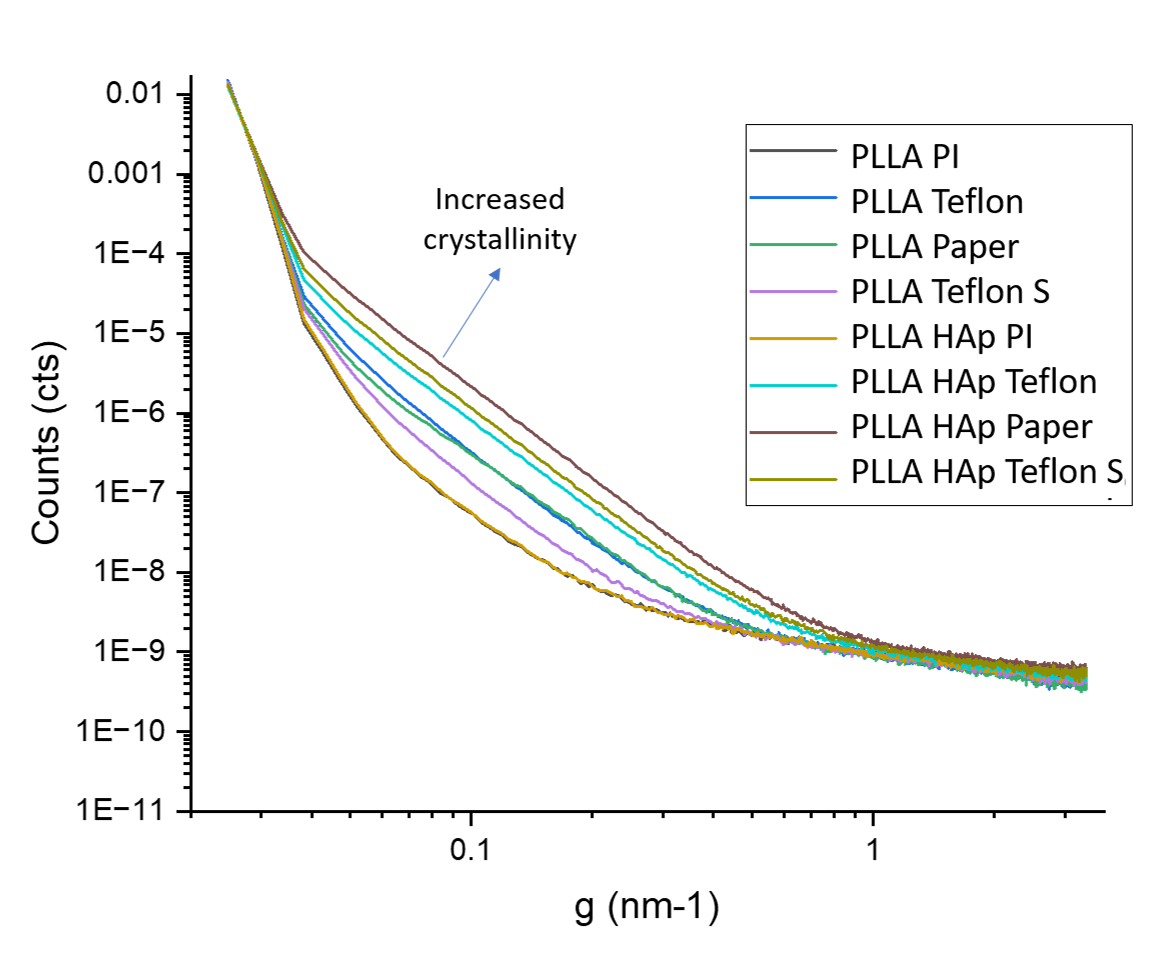

Supplement: Supplementary file 1 [file polymers-18-00257-s001.zip › Figure S1.tif]

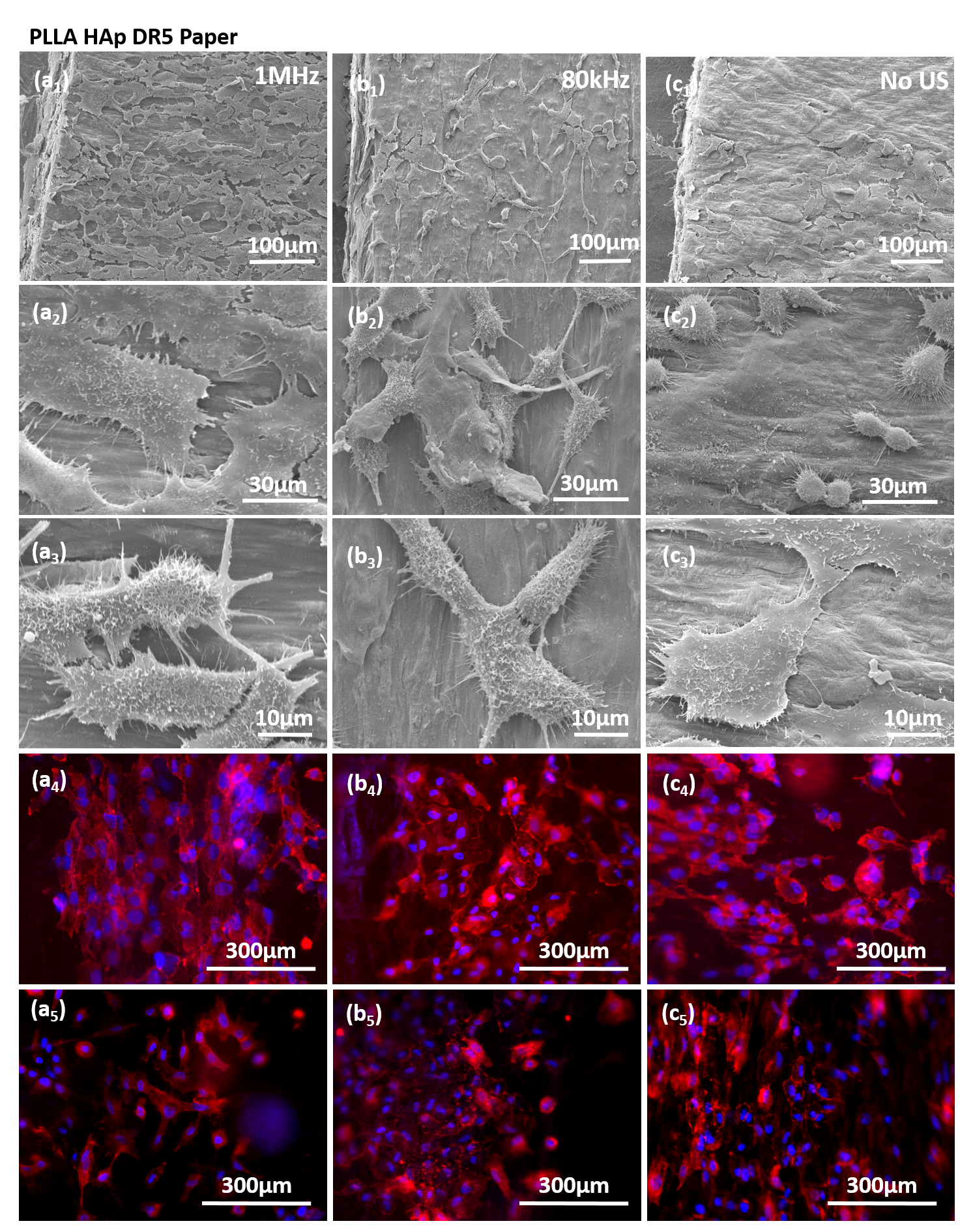

Supplement: Supplementary file 1 [file polymers-18-00257-s001.zip › Figure S2.tif]

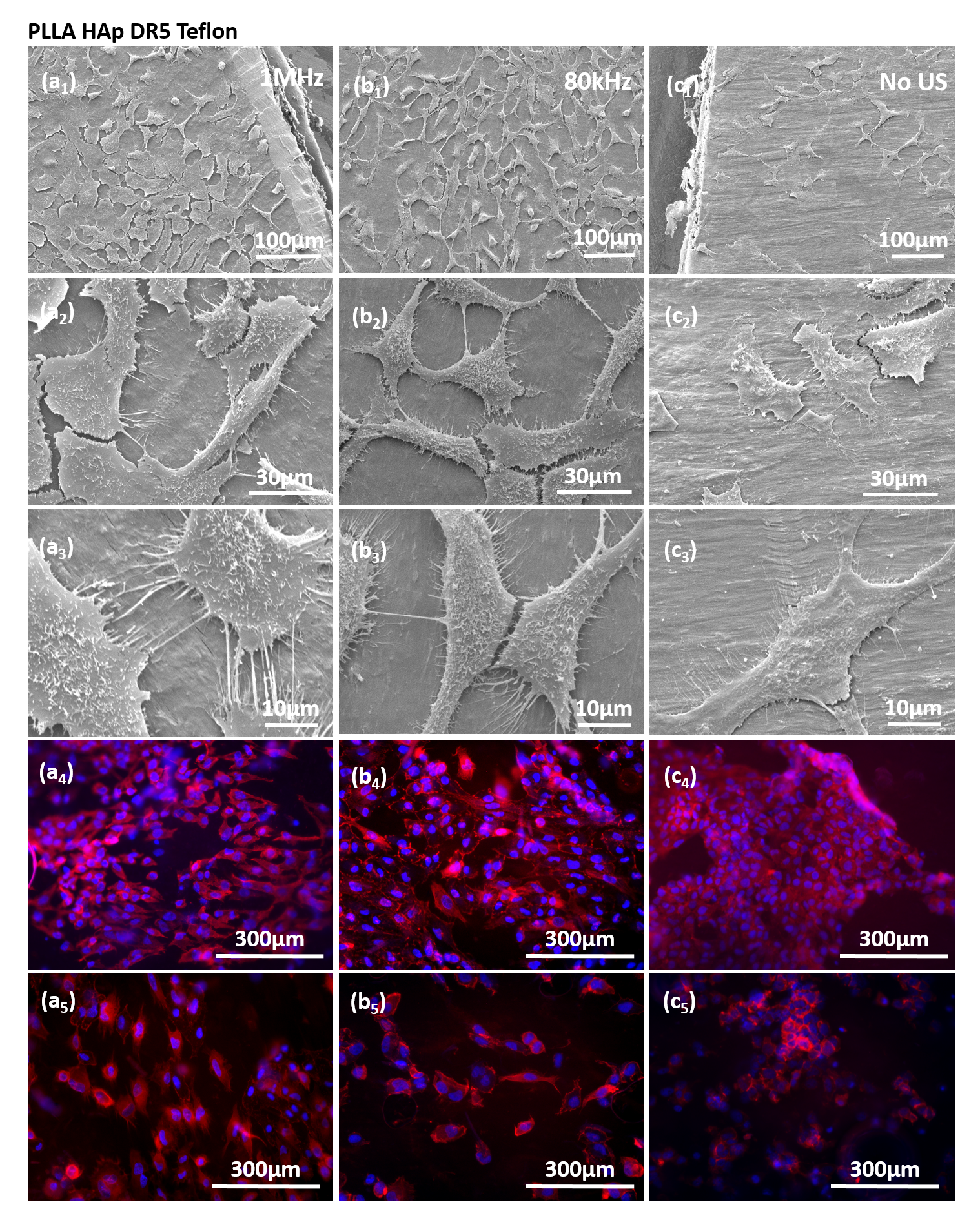

Supplement: Supplementary file 1 [file polymers-18-00257-s001.zip › Figure S3.tif]

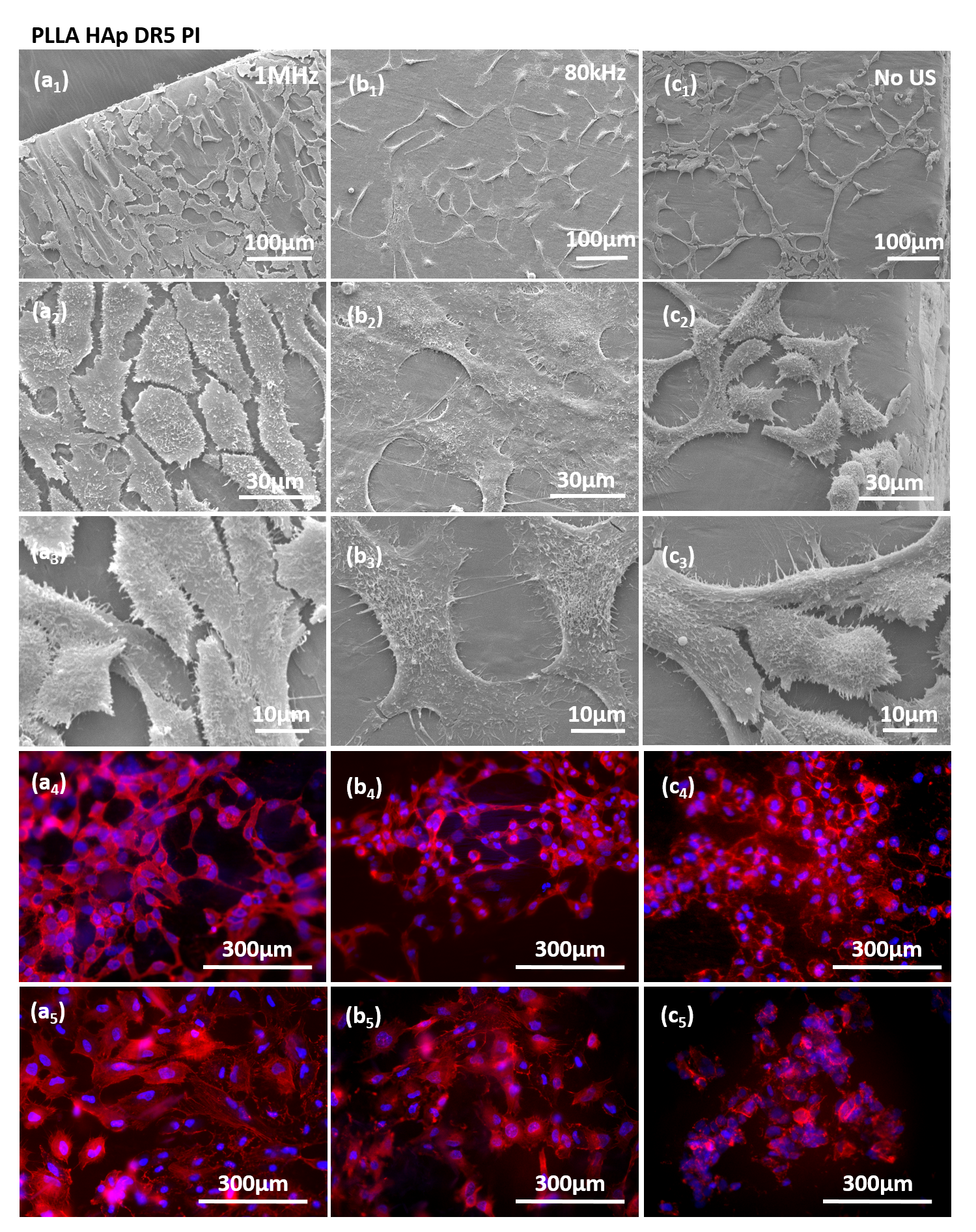

Supplement: Supplementary file 1 [file polymers-18-00257-s001.zip › Figure S4.tif]
